# Supplementary material for: Genome-Wide Association Study Identifies Candidate Genes Associated with Feet and Leg Conformation Traits in Chinese Holstein Cattle
Source: Animals (Basel). 2021 Jul 30;11(8):2259. doi: 10.3390/ani11082259 (PMC8388412; doi:10.3390/ani11082259)
Supplement: Supplementary file 1 [file animals-11-02259-s001.zip › Supplementry file/Figure S1. Data Quality Control.pdf]

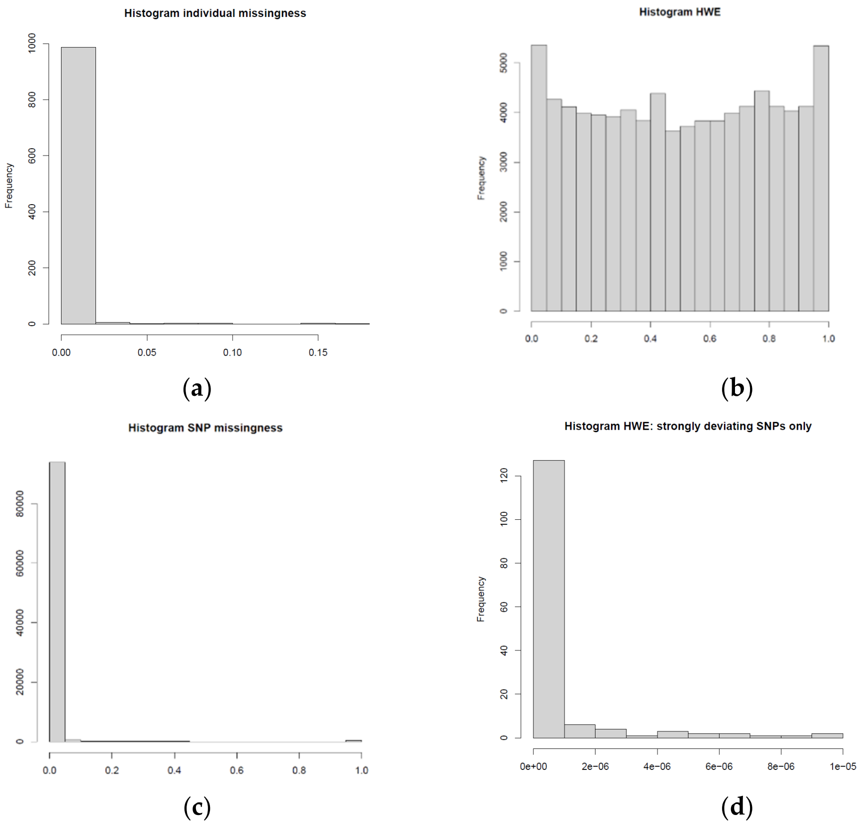


**Figure S1.** Data Quality Control: (**a**) Histogram of individual filtering (**b**) Histogram of SNP filtering (**c**) Hardy–Weinberg equilibrium (HWE) (**d**) Histogram SNP divination from HWE
